# Usefulness of ^18^F-fluorodeoxyglucose-positron emission tomography/computed tomography in primary cystadenocarcinoma of the mesentery: a case report

**DOI:** 10.1186/s40792-020-01079-2

**Published:** 2020-12-04

**Authors:** Yuto Hozaka, Yuko Mataki, Hiroshi Kurahara, Kiyonori Tanoue, Tetsuya Idichi, Yota Kawasaki, Satoshi Iino, Pramod Nepal, Takaaki Arigami, Kosei Maemura, Hirotsugu Noguchi, Hiroyuki Shinchi, Akihide Tanimoto, Shoji Natsugoe, Takao Ohtsuka

**Affiliations:** 1 Department of Digestive Surgery, Breast and Thyroid Surgery, Graduate School of Medical and Dental Sciences, Kagoshima University, 8-35-1, Sakuragaoka, Kagoshima, 890-8520 Japan; 2 Department of Pathology, Graduate School of Medical and Dental Sciences, Kagoshima University, Kagoshima, Japan; 3 Department of Health Sciences, School of Medicine, Kagoshima University, Kagoshima, Japan

**Keywords:** Mesenteric cyst, Cystadenoma, Cystadenocarcinoma, Mesentery, Mesocolon, PET, PET/CT

## Abstract

**Background:**

Mesenteric cysts have various histological forms, including mesenteric cystadenomas and borderline cystic neoplasms. Primary cystadenocarcinoma of the mesentery is extremely rare; therefore, the clinical and radiological features of this tumor have not been fully elucidated.

**Case presentation:**

A 50-year-old Japanese woman had a complaint of a left-sided abdominal distention. Enhanced computed tomography and magnetic resonance imaging revealed a unilocular cystic lesion measuring approximately 10 cm located in the left side of the abdomen. ^18^F-fluorodeoxyglucose (FDG)-positron emission tomography/computed tomography (PET/CT) revealed mottled mild FDG uptake in the cyst wall and intense FDG uptake in several mural nodules. The cystic mass with the descending colon was completely removed. Pathological examination of the specimens revealed various histologic patterns of adenocarcinoma, including mucin production in the mural nodules. We eventually diagnosed a primary cystadenocarcinoma arising from the mesentery of the descending colon.

**Conclusions:**

Malignancy should be suspected in mesenteric or retroperitoneal cystic tumors with high FDG uptake, and complete resection should be performed with adequate margins.

## Background

Mesenteric cysts are relatively rare and have various histological forms [1–3]. Mesenteric cysts include mesenteric cystadenomas, borderline cystic neoplasms, and mesenteric cystadenocarcinomas that are similar to ovarian tumors [3–5]. Since primary cystadenocarcinoma is extremely rare, the differences in clinical and radiological features among benign, borderline, and malignant lesions are unclear [4]. Here, we present a case of mesenteric cystadenocarcinoma suspected to be a malignant tumor based on preoperative positron-emission tomography/computed tomography (PET/CT) findings.

## Case presentation

**Fig. 1:**
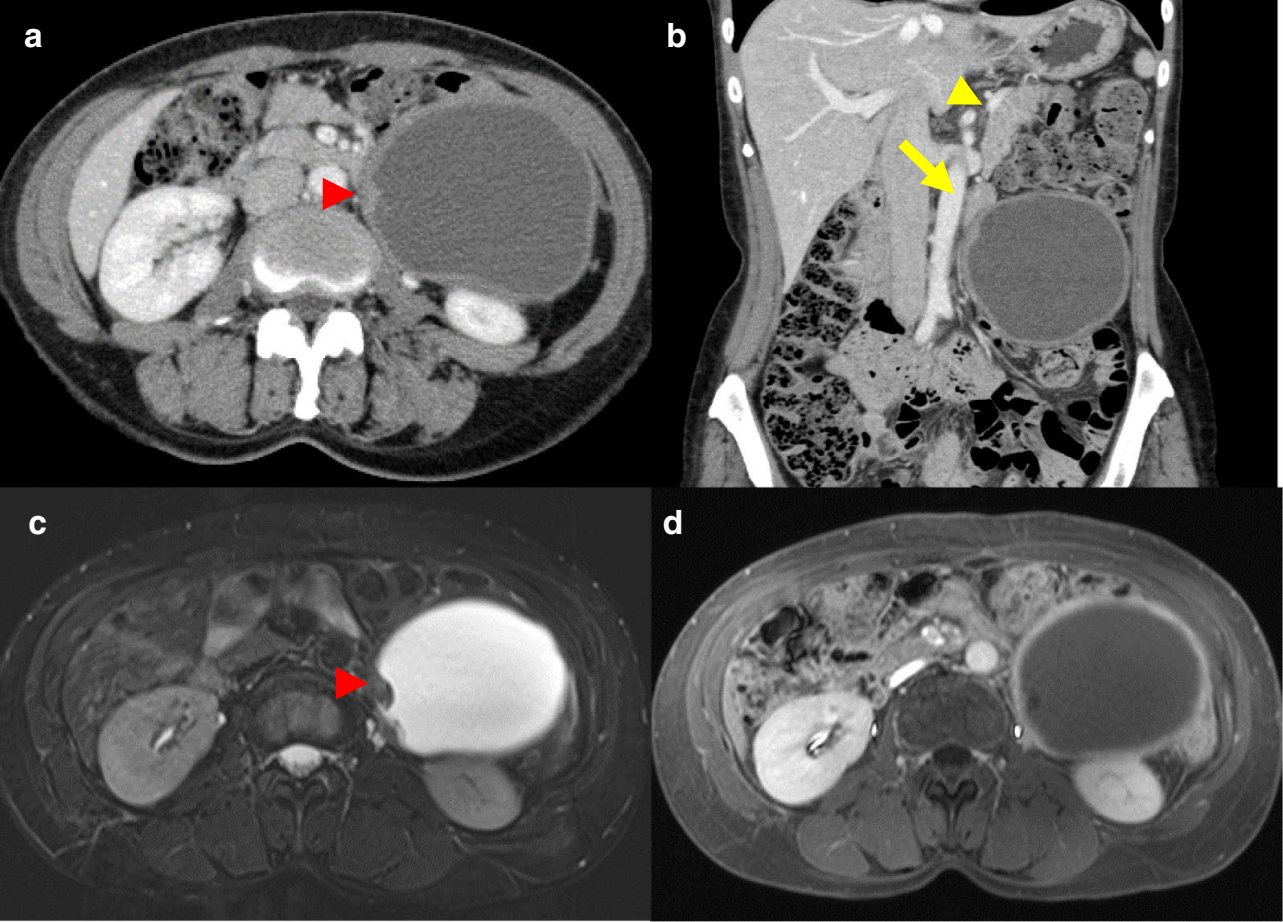
Preoperative abdominal contrast-enhanced computed tomography (CT) and magnetic resonance imaging (MRI) findings. **a** CT revealing a large unilocular cystic mass with three mural nodules (red arrow’s head) in the left retroperitoneal space on axial view. The boundary between the nodule and the wall was unclear in all three nodules. **b** CT demonstrating that the cystic mass was located close to the proximal side of the jejunum (yellow arrow) and the mass was separated from kidney and pancreas (yellow arrow’s head) on coronal view. **c** T1-weighted images revealing low signal intensity of the cystic wall, including mural nodules, and high signal intensity of the cyst component on axial view. In contrast to CT, mural nodules were clearly delineated at the rise. **d** T2-weighted images revealing high signal intensity of the cystic wall including mural nodules and low signal intensity of the cyst component on axial view

**Fig. 2:**
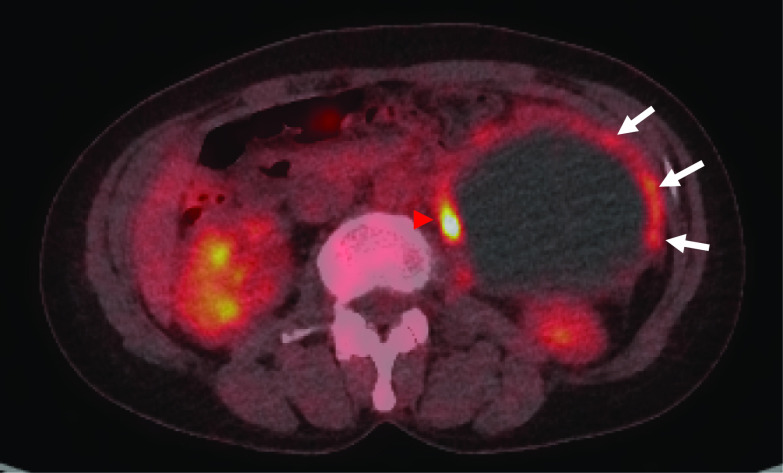
Preoperative 18 F-fluorodeoxyglucose (FDG)-positron emission tomography/computed tomography (PET/CT) findings. FDG-PET/CT revealed mottled FDG uptake in the whole cyst wall (white arrows) and intense FDG uptake of several mural nodules (red arrow’s head)

A 50-year-old Japanese woman presented to a hospital with left-sided abdominal distention. Abdominal ultrasonography revealed a cystic mass in the left-lower quadrant of the abdomen measuring approximately 10 cm in diameter. The patient had a history of iron deficiency anemia due to menorrhagia. She was referred to our hospital for surgical management. Upon admission, she complained of left-sided abdominal distention and denied abdominal pain and nausea. Physical examination revealed mild distention and a mildly tender mass in the left-lower quadrant of the abdomen. Laboratory examination findings on admission revealed elevated serum tumor marker levels: carcinoembryonic antigen (CEA) level of 21.4 ng/mL (normal range: 0–5.0) and carbohydrate antigen 19-9 (CA19-9) level of 804.0 U/mL (normal range: 0–37.0). Other laboratory results were normal except for a low hemoglobin level of 10.4 g/dL. Abdominal US revealed a tumor located in the left abdomen with a maximum diameter of about 9 cm. The cyst wall was about 4 mm thick and relatively uniform, with no septa or nodules visible within the cyst. Enhanced computed tomography (CT) and magnetic resonance imaging (MRI) of the abdomen revealed a 10.2 × 9.1 × 9.3-cm-sized unilocular cystic lesion with maximum 12-mm-sized nodules located on the left side of the abdomen (Fig. 1a–d). CT revealed three uniform contrast-enhanced nodules similar to the cyst wall. The boundary between the nodule and the wall was unclear for all three nodules; one of the nodules was seen as a gentle ridge. MRI revealed three nodules in the cyst, and the nodules and cyst wall showed low signal intensity on T1-weighted images and high signal intensity on T2-weighted images. The cystic mass was close to the left ureter, proximal jejunum, and descending colon, but separated from the left kidney and pancreas. The cyst wall was 3-mm thick. ^18^F-fluorodeoxyglucose (FDG)-PET/CT revealed mottled mild FDG uptake in the whole cyst wall and intense FDG uptake in the mural nodules with standardized uptake value (SUV)-max of 16.15, and no abnormal FDG uptake in other organs (Fig. 2). There were no malignant tumors on endoscopic gastrointestinal examination, and no findings to suspect gynecological diseases such as ovarian tumors. Although we were not able to identify the exact origin of the cyst based on radiological findings alone, we suspected a primary malignant cystic tumor arising from the retroperitoneum.

**Fig. 3:**
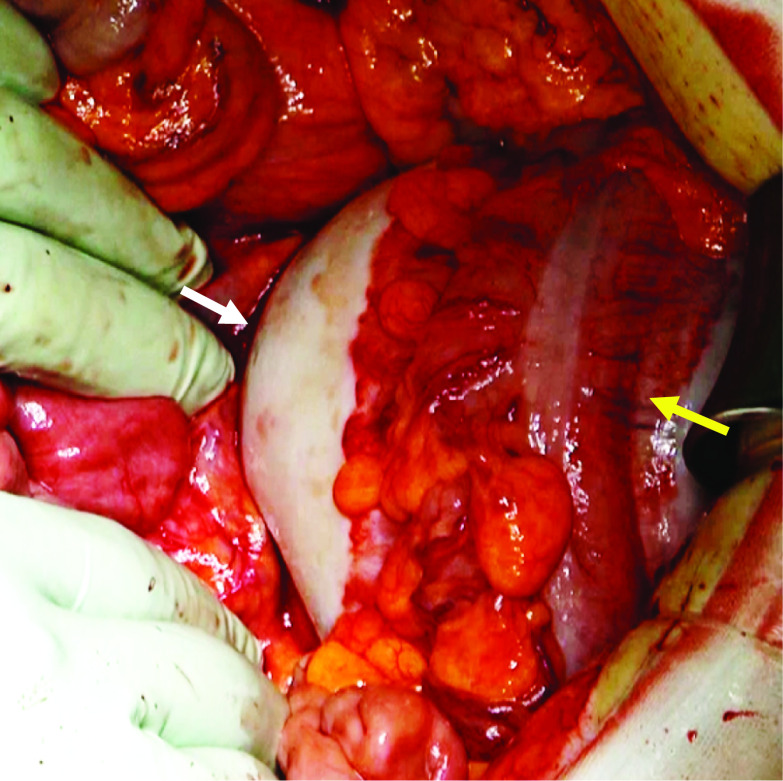
Intra-operative findings. The cystic mass (white arrow) located in the mesentery of the descending colon was rigidly attached to the descending colon (yellow arrow) but was not adhered to any other organs

A midline laparotomy was performed after confirming the absence of disseminated lesions by laparoscopy and it revealed a cystic mass located in the mesentery of the descending colon (Fig. 3). The cystic mass was rigidly attached to the descending colon, but not adhered to any other organs. The arterial supply of the tumor originated from the left colic artery; based on this, we diagnosed a mesenteric cystic tumor rather than a retroperitoneal cystic tumor. The cystic mass with the descending colon were completely removed without rupture.

**Fig. 4:**
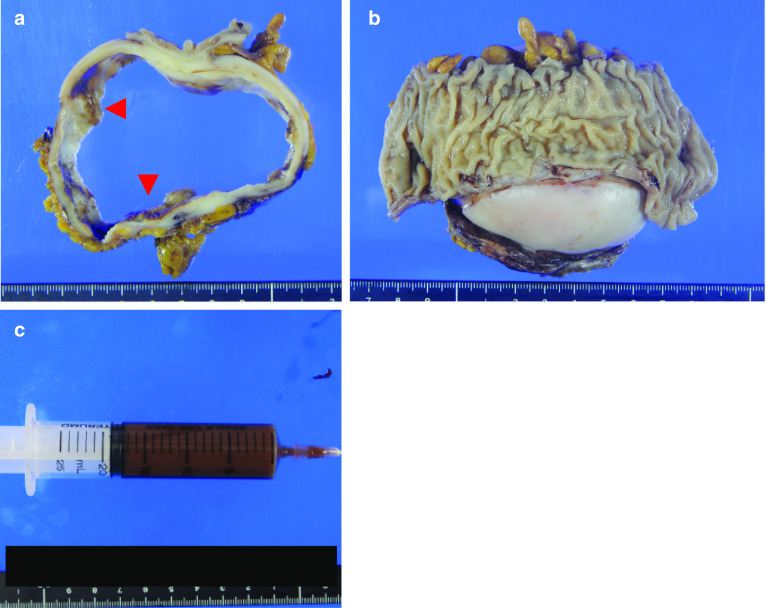
Gross findings. **a** Split findings of the resected specimens revealing that the tumor was an unilocular cystic mass, and the cyst wall had uneven wall thickness and had some raised nodules (red arrowheads). **b** The findings of the resected specimens revealed no obvious tumor in the mucosa of the descending colon. **c** The cyst component contained a serous cloudy ‘café-au-lait-like’ fluid

**Fig. 5:**
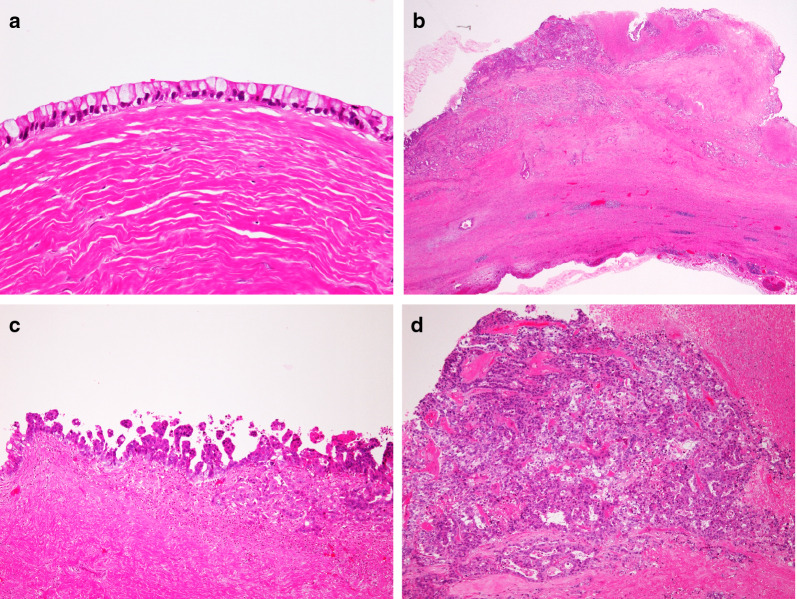
Histopathological findings. **a** Microscopic findings (hematoxylin and eosin staining; magnification, ×400) reveal a cyst wall mainly lined with mucinous columnar epithelium. **b**–**d** In the nodule, there were various histologic patterns of adenocarcinoma including irregular glandular, sheet-like structures, and the surface was covered with papillovillous component. The microscope magnification is ×20 in **b**, ×100 in **c**, and ×100 in **d**

**Fig. 6:**
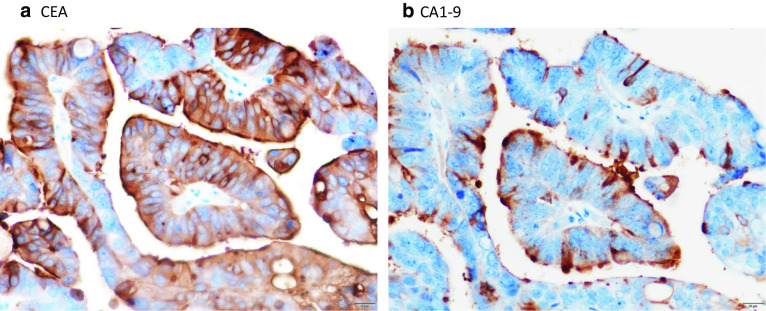
Immunohistochemical images with staining of CEA and CA19-9. **a** Immunohistochemical images with staining of CEA (magnification, ×400) revealed overexpression of CEA was detected inside the carcinoma cell of the mesenteric cystadenocarcinoma. **b** Immunohistochemical images with staining of CA19-9 (magnification, ×400) revealed CA19-9 was detected mainly on cell membranes of the carcinoma. Representative image was in the mural nodule

The surgical specimen measured 9.2 × 9.2 × 6.5 cm (Fig. 4a, b). The cyst contained a serous, cloudy, ‘café-au-lait-like’ fluid (Fig. 4c). The pathological diagnosis of the specimen was adenocarcinoma. However, it was difficult to determine the histopathological subtypes (mucinous, serous, or seromucinous) due to the atypical histologic findings. The inner surface of the cyst wall was lined predominantly by a single layer to multiple layers of columnar epithelium (Fig. 5a) and partially by simple cuboidal epithelium. The nodules consisted of atypical cells that showed nuclear atypia and abundant clear or eosinophilic cytoplasm composed of an irregular glandular or sheet-like structure with stromal infiltration (Fig. 5b, c), and the surface was covered with papillovillous component (Fig. 5d). The mesenteric tumor slightly invaded the subserosal layer of the descending colon, but not the mucosal layer. No ovarian stroma or teratomatous elements, including ectopic endometriosis, were observed. The atypical cells were immunohistochemically reactive for cytokeratin 7 (CK7), and p53, but negative for cytokeratin 20 (CK20), estrogen receptor (ER), progesterone receptor (PgR), Wilms’ tumor gene-1, napsin A, and calretinin. Therefore, the final diagnosis was primary cystadenocarcinoma arising from the mesentery of the descending colon; the atypical cells were immunohistochemically reactive for CEA and CA19-9 (Fig. 6a, b).

The postoperative course was uneventful, and the patient was discharged 11 days after the surgery. Adjuvant chemotherapy was initiated with S-1 (TS-1; tegafur, gimeracil, and oteracil potassium) at 100 mg/kg body weight per day for 8 courses (4 weeks of administration and 2 weeks of discontinuation). Approximately 1 year after the surgery, abdominal CT revealed no signs of disease recurrence, and serum CEA and CA19-9 levels had returned to normal.

## Discussion

Mesenteric cysts are lesions that occur in or near the mesentery; they are not parasitic or dermoid and are not derived from normally placed retroperitoneal organs [6]. Mesenteric cysts are rare, with an incidence of 1/27,000 to 1/250,000 [7]. Among mesenteric cysts, primary cystadenocarcinoma is extremely rare, and therefore the clinical features of benign, borderline, and malignant primary cystadenomas are unclear and have not been fully differentiated [4]. Furthermore, it has been reported that the preoperative diagnosis for benign or malignant mesenteric cyst by routine imaging modalities is difficult, and the usefulness of PET/CT for this purpose is not well understood [1].

Thus, we performed a search of studies published in English between 1933 and August 2020 and reviewed the clinicopathologic features of cystadenomas, borderline malignant cystic neoplasms, and cystadenocarcinomas. Cases of retroperitoneal cysts and adenocarcinomas without cystic lesions were excluded. After excluding cases with insufficient information, a total of 14 cases of primary benign cystic neoplasms (cystadenomas) and 6 cases of borderline malignant cystic neoplasms were identified [3, 5, 8–23]. We also identified 9 cases of primary cystadenocarcinoma of the mesentery, including the current case (Table 1) [4, 6, 24–29].

**Table 1:** Summary of clinicopathological features of cystic neoplasm cases of the mesentery

Characteristics	Cystadenoma n = 14	Borderline malignant cystic neoplasm n = 6	Cystadenocarcinoma of the mesentery n = 9
Sex			
Female	12	5	8
Male	1	1	1
Age (years)			
Median	43 (14–80)	38 (32–54)	41 (23–72)
Symptoms and signs (including duplicate)			
Incidental finding	5	0	1
Abdominal pain/back pain	4	3	3
Abdominal distention/discomfort	4	5	2
Nausea/vomiting	0	2	1
Weight loss	0	0	2
Not described	2	1	0
Preoperative imaging modality (including duplicate)			
US	2	4	3
CT	7	4	7
MRI	7	2	1
PET/CT	0	0	1
Not described	2	–	–
Tumor location			
Appendix	1	0	0
Ascending colon	1	1	1
Transverse colon	1	0	3
Descending colon	2	2	3
Sigmoid colon	4	1	2
Sigmoid and descending colon	0	1	0
Small intestine	3	0	0
Mesentery (not described)	2	1	0
Tumor size (Maximum diameter: cm)			
Median	12 (7–40)	17 (10–25)	10 (5–18)
Internal structure			
Unilocular	3	2	2
Multilocular	4	2	2
Not described	7	2	5
Surgical treatment			
Only cystectomy	6	1	3
Cystectomy with bowel resected	5	1	3
Cystectomy with combined resection involving other organs	1	1	1
Cytoreductive surgery	0	0	1
Unresectable	0	0	1
Not described	2	3	0
Subtype of final diagnosis			
Mucinous	14	6	3
Serous	0	0	2
Seromucinous	0	0	1
Indistinguishable	0	0	3

The median ages of patients with benign cystadenoma, borderline malignant cystic neoplasm, and cystadenocarcinoma of the mesentery were 43 (14–80) years, 38 (32–54) years, and 41 years (23–72 years), respectively. There was no significant difference in age between patients with benign and malignant tumors. These tumors were more common in women, and only 3 cases have been reported in men [16, 22, 29]. Regarding the subtype of the final diagnosis, benign cystadenoma and borderline cystic neoplasm were all mucinous. On the other hand, cystadenocarcinoma was mucinous in 3 cases, serous in 2 cases, and seromucinous in 1 case. In the current case, mucin-producing cells were primarily present in the cyst wall and in the adenocarcinoma; however, the cyst wall was partially contained in serous-like cuboidal cells. The morphology of the ductal structure was diverse. Thus, it was difficult to diagnose mucinous, serous or seromucinous adenocarcinoma, and the diagnosis was only cystadenocarcinoma.

Regarding preoperative imaging modality, in our literature review, US was more frequently used before 2000, whereas CT was used more frequently than after 2000, and MRI findings are gradually being reported. CT and MRI can confirm the positional relationship between the cystic tumor and adjacent organs, which is useful for estimating the origin. Although it has been speculated that the amount of soft tissue and internal septations may suggest malignancy, as with cystic tumors of the ovary, in this review, there were only two cases each with solid components in cystadenoma and cystadenocarcinoma. There were no reports containing information on internal septation [30, 31]. To the best of our knowledge, there are no typical findings indicative of malignant mesenteric tumors; none of the cases had a preoperative diagnosis of mesenteric cystic neoplasm based on radiological imaging. Knezevic et al. reported that retroperitoneal cysts had no specific radiological features, which made obtaining an accurate preoperative diagnosis with standard imaging modalities nearly impossible [29]. Preoperative differential diagnosis may also be difficult for mesenteric cysts that have the same developmental origin as retroperitoneal cysts and are grouped into a single entity.

In the current case, we considered the possibility of a malignant cystic tumor based on the high serum tumor marker levels and high uptake of FDG in mural nodules on FDG-PET/CT. FDG-PET/CT provides important physiologic information on altered tissue metabolism that forms the basis for detection and diagnosis of carcinoma. Compared to normal cells, malignant cells develop significant alterations in carbohydrate metabolism [32]. Additionally, PET/CT is useful for staging, optimizing treatment, restaging, therapy monitoring, and prognostication of various types of malignant tumors [33]. To our knowledge, there are no reports of the use of PET/CT for mesenteric cystadenocarcinoma. Furthermore, only a few cases on the use of FDG-PET/CT for evaluation of retroperitoneal tumors have been reported to date, with 2 cases of benign mesenteric cysts, 1 case of a retroperitoneal cystadenocarcinoma, and 1 case of retroperitoneal cystic tumor [33–36]. The morphology of pancreatic cystic tumor and ovarian tumors is similar to that of cystadenoma and cystadenocarcinoma. Sperti et al. demonstrated the importance of FDG accumulation in identifying malignant cystic tumors of the pancreas [37]. Approximately 94% of malignant cystic tumors showed FDG uptake with SUV of 2.6 to 12.0; only 1 in 39 cases of mucinous cystadenoma showed increased SUV of 2.6. The sensitivity, specificity, and positive and negative predictive values for FDG-PET in detecting malignant tumors were 94%, 97%, 94%, and 97%, respectively. Although the study did not mention the relationship between the site of accumulation and the tumor, high SUV in cystic tumor may be highly indicative of carcinoma. However, it should be kept in mind that FDG not only accumulates in well-metabolized tumors like carcinoma, but also in inflammatory areas [38, 39]. In the current case, there were no inflammatory findings, including cystic tumor, detected preoperatively. We were able to make one-on-one comparison between the uptake areas in PET/CT and the pathological findings. The FDG accumulation site contained more adenocarcinoma than other areas, highly suggesting that the site of FDG accumulation was not due to inflammation but as a result of adenocarcinoma. If FDG-PET/CT reveals high FDG uptake in the absence of inflammatory findings, the possibility of malignancy should be considered and appropriate treatment should be initiated [33–36]. It is difficult to distinguish benign from malignant tumors using standard radiological imaging such as CT, MRI, and US. In comparison, FDG-PET/CT may be useful in differentiating mesenteric cystic neoplasm, since in the current case, FDG accumulation was witnessed in adenocarcinoma.

The recommended treatment for cystadenomas is surgical resection to obtain a histopathological diagnosis and manage tumor-related symptoms [4, 28, 40]. There have been no cases of lymph node metastasis from mesenteric cystadenocarcinoma; postoperative local recurrence, peritoneal dissemination recurrence, and even benign peritoneal pseudomyxoma have been reported [28, 40]. Therefore, although there is no evidence for active lymph node dissection, complete resection with adequate margin to prevent local recurrence or pseudomyxoma recurrence should be undertaken. Although there is inadequate evidence regarding the use of adjuvant chemotherapy for mesenteric cystadenocarcinoma, we decided on oral administration of S-1, considering the risk of recurrence. S-1 is considered to be highly versatile and is indicated for adenocarcinomas in various organs, including gastrointestinal cancers [41, 42].

## Conclusions

We report the first case of primary cystadenocarcinoma of the mesentery in which FDG-PET/CT was performed preoperatively. If high FDG is accumulated in the mesenteric or retroperitoneal cystic tumor, we should consider the possibility of malignancy and perform complete resection with adequate margins.

## Data Availability

The datasets supporting the conclusions of this article are included within the article and its additional files.

## Abbreviations

PET/CT: Positron emission tomography/computed tomography
CEA: Carcinoembryonic antigen
CA19-9: Carbohydrate antigen 19-9
CT: Computed tomography
MRI: Magnetic resonance imaging
FDG: ^18^ F-fluorodeoxyglucose
SUV: Standardized uptake value
CK7: Cytokeratin 7
CK20: Cytokeratin 20
ER: Estrogen receptor
PgR: Progesterone receptor
